# Association of a genetic polymorphism (-44 C/G SNP) in the human *DEFB1 *gene with expression and inducibility of multiple β-defensins in gingival keratinocytes

**DOI:** 10.1186/1472-6831-9-21

**Published:** 2009-08-27

**Authors:** Andrea A Kalus, L Page Fredericks, Beth M Hacker, Henrik Dommisch, Richard B Presland, Janet R Kimball, Beverly A Dale

**Affiliations:** 1 Department of Medicine/Dermatology, University of Washington, Seattle, Washington 98195, USA; 2 Department of Oral Biology, University of Washington, Seattle, Washington 98195, USA; 3 Veterinary Clinical Medicine Department, University of Illinois at Urbana-Champaign, Urbana, IL 61802, USA; 4 Poliklinik für Parodontologie, Zahnerhaltung und Präventive Zahnheilkunde, Universitätsklinikum Bonn, Welschnonnenstrasse 17, 53111 Bonn, Germany

## Abstract

**Background:**

Human β-defensins (hBDs) are antimicrobial peptides with a role in innate immune defense. Our laboratory previously showed that a single nucleotide polymorphism (SNP) in the 5' untranslated region of the hBD1 gene (*DEFB1*), denoted -44 (rs1800972), is correlated with protection from oral *Candida*. Because this SNP alters the putative mRNA structure, we hypothesized that it alters hBD1 expression.

**Methods:**

Transfection of reporter constructs and evaluation of antimicrobial activity and mRNA expression levels in keratinocytes from multiple donors were used to evaluate the effect of this SNP on constitutive and induced levels of expression.

**Results:**

Transfection of CAT reporter constructs containing the 5' untranslated region showed that the -44 G allele yielded a 2-fold increase in CAT protein compared to other common haplotypes suggesting a *cis *effect on transcription or translation. The constitutive hBD1 mRNA level in human oral keratinocytes was significantly greater in cells from donors with the -44 GG genotype compared to those with the common CC genotype. Surprisingly, the hBD3 mRNA level as well as antimicrobial activity of keratinocyte extracts also correlated with the -44 G allele. Induced levels of hBD1, hBD2, and hBD3 mRNA were evaluated in keratinocytes challenged with Toll-like receptor 2 and 4 ligands, interleukin-1β, TNFα, and interferon-γ (IFNγ). In contrast to constitutive expression levels, IFNγ-induced keratinocyte hBD1 and hBD3 mRNA expression was significantly greater in cells with the common CC genotype, but there was no clear correlation of genotype with hBD2 expression.

**Conclusion:**

The *DEFB1 *-44 G allele is associated with an increase in overall constitutive antimicrobial activity and expression of hBD1 and hBD3 in a manner that is consistent with protection from candidiasis, while the more common C allele is associated with IFNγ inducibility of these β-defensins and is likely to be more protective in conditions that enhance IFNγ expression such as chronic periodontitis. These results suggest a complex relationship between genetics and defensin expression that may influence periodontal health and innate immune responses.

## Background

The health of mucosal surfaces, the oral cavity, and skin is maintained by a complex balance among commensal and pathogenic organisms and host defenses [1-4]. Epithelial cells express multiple antimicrobial peptides that act as a first line of defense and help to keep these microbes in balance (reviewed by Dale and Fredericks [5]). In addition to functioning as antimicrobial agents, these peptides have immunomodulatory properties and serve as links between the innate and acquired immune systems. For example, the β-defensins act as chemokines and signaling molecules to antigen presenting cells [6,7] and thus function more broadly in host defense as initiators of the acquired immune system.

Over 30 human β-defensins genes have been identified [8], however most studies have focused on hBD1, -2, and -3, which are expressed in the oral cavity [9-14], skin [15], gut and other epithelia [16-18]. HBD2 and hBD3 are induced by various bacterial components and cytokines, while hBD1 is constitutively expressed [5]. Nevertheless, the level of hBD1 expression varies among individuals and it has been suggested that this variation is due to genetic differences in the *DEFB1 *gene encoding hBD1 [12,13,19]. This type of individual variation is important in the functional diversity that underlies the regulation of innate immune function and is evident in many components of innate immunity [20].

Growing evidence links antimicrobial peptides with resistance or susceptibility to mucosal infections and disease susceptibility [18,21-23]. For example, levels of hBD2 and LL-37 are low in individuals with atopic dermatitis [24] and LL-37 and α-defensins are poorly expressed in a severe genetic form of periodontal disease [25]. Two types of genetic polymorphisms have been identified in the genes encoding defensins, copy number polymorphisms (CNPs) and single nucleotide polymorphisms (SNPs). Copy number polymorphism in the α-defensin gene cluster is associated with expression level in neutrophils [26,27]. Low expression of both α – and β-defensins are associated with specific forms of Crohn's disease and low β-defensin copy number increases susceptibility to colonic Crohn's disease [28,29]. Multiple SNPs within the *DEFB1 *gene [30-33] have also been associated with health risks. Previous work in our laboratory showed that a C → G SNP in the *DEFB1 *5' untranslated region (UTR) at position -44 relative to the AUG protein initiation site (SNP rs1800972) is correlated with low oral levels of the common yeast, *Candida albicans *[34]. This SNP is also associated with protection from chronic obstructive pulmonary disease [35] and vertical transmission of HIV infection [36-38], as well as Crohn's disease [39]. In the skin this SNP is associated with atopic dermatitis [40] while high copy number in the β-defensin gene cluster increases the risk for psoriasis [41].

The objective of this study was to examine the association of the *DEFB1 *-44 SNP and expression of hBD1 in oral keratinocytes. We used two independent methods to evaluate the effect of this SNP on constitutive levels of protein and/or mRNA expression as well as the antimicrobial activity of epithelial cell extracts. We also evaluated the association of this SNP with expression of constitutive and induced levels of hBD2 and hBD3 which are encoded by genes located in a cluster near the *DEFB*1 gene on chromosome 8p22–23 [42]. Our results show that the -44 SNP is associated with increased expression of hBD1 and hBD3 and that the *DEFB1 *-44 SNP has a complex effect on expression and inducibility of multiple β-defensins.

## Methods

### Human oral keratinocytes (HOKs)

Human oral keratinocytes (HOKs) were prepared from multiple individual donors in accordance with an approved University of Washington Institutional Review Board protocol. Oral cells were obtained and cultured as previously described [12]. Briefly, primary epithelial cells from gingival tissue were grown in keratinocyte basal medium (KBM, Cambrex Bioscience, Walkersville, MD) with keratinocyte growth medium (KGM) supplements in 0.03 mM Ca^2+ ^for three passages, then raised to either 0.15 mM or 0.6 mM Ca^2+ ^to induce differentiation. Cells were harvested for DNA or RNA after growth for an additional 48 hr and harvested for protein after 96 hr as described below. For induced cultures cells at 80% confluence were stimulated with 100 ng/ml final concentration of *Eschericia coli *lipopolysaccharide (LPS) (Sigma, St. Louis, MO), PAM3CSK4 (Invitrogen, San Diego, CA), interleukin1β (IL1β, Cell Sciences, Canton, MA), TNFα (Cell Sciences), and interferon-γ (IFNγ, Cell Sciences) for 18 hr in 0.15 mM Ca^+2 ^then harvested for RNA, The *E. coli *serotype 0127:B8 LPS was further purified by phenol extraction as previously described [43].

### Genotyping

Genomic DNA was extracted from HOKs (DNeasy tissue kit, Qiagen, Valencia, CA), and the concentration measured by Nanodrop absorbance (ThermoScientific, Wilmington, DE). The hBD1 5' UTR was sequenced in the Dept. of Genome Sciences, University of Washington, courtesy of Dr. Robert Livingston or by Polymorphic DNA Technologies Inc (Alameda, CA) using primers specific for exon 1 (Table 1).

**Table 1:** Oligonucleotide PCR primers utilized

**Name**	**Sequence**		**Anneal. Temp**.
**DNA Sequencing**			
HBD1 SEQ	AACTCTAGCAGGTACCAGAGCTTACCT		65
	CTAACCTAGAAAACCAAACAGGAGGAG		
DEFB1 SNEST	TGAGGCCATCTCAGACAAAA		65
	GCTCCAGGCGTAAAGCTAAA		
			
**QPCR**		**Primer Efficiency**	

HBD1	CACTTGGCCTTCCCTCTGTA	1.91	56
	CGCCATGAGAACTTCCTACC		
HBD2	GGAGCCCTTTCTGAATCCGCA	1.98	65
	CCAGCCATCAGCCATGAGGGT		
HBD3	GTGAAGCCTAGCAGCTATGAGGAT	1.68	60
	TGATTCCTCCATGACCTGGAA		
RPO	GCCTTGACCTTTTCAGCAAG	2.04	59
	GCAGCATCTACAACCCTGAAG		
Beta-actin	CTCTTCCAGCCTTCCTTCCT	1.75	63
	AGCACTGTGTTGGCGTACAG		

### Putative RNA folding

The hBD1 mRNA folding characteristics were estimated using the MFOLD program (Rensselaer Polytechnic Institute, Troy, NY) [44].

### Plasmid constructs

We prepared four constructs containing the most common haplotypes of the three known hBD1 5' UTR SNPs (-20, -44, -52) [31] (GenBank accession no. U50930; rs 11362, 1800972, 1799946) upstream of the chloramphenicol acetyltransferase (CAT) reporter gene using the pc DNA3CAT plasmid (Invitrogen, Carlesbad, CA). For details of plasmid construction see Additional file 1. A construct with the CMV 5' UTR and a promoterless pGEM-NCMV were created and served as controls. pKTCAT is a promoterless CAT reporter plasmid that contains the BamHI-Hind III CAT fragment of pSV0 CAT cloned into the AatII-Hind III sites of pUCl8 [45]. pcDNA3CAT is a positive control CAT plasmid. The constructs with the common haplotypes were: pGEM-ACG (-20 = A, -44 = C, -52 = G), pGEM-GCA and pGEM-GGG; an additional construct, pGEM-GCG, was also prepared as a direct control for pGEM-GGG, even though this haplotype has not been observed in the general population [31].

### Cell culture and transfections

COS-7 cells were grown and transfected as previously described [46] using Lipofectamine Plus reagent (Invitrogen) at 20 μg/ml. Cells were transiently transfected with 1 μg of each of the five pGEM constructs, pcDNA3CAT, or pKCAT (a promoterless pcDNA3CAT), and co-transfected with pSVβ-gal (Promega, Madison, WI) to allow normalization of transfection efficiency. After 4 hr, fetal bovine serum was added to a final concentration of 10%. Cells were harvested after 48 hr.

### CAT and β-galactosidase assays

Two methods were used to measure CAT reporter protein expression, a radioactive enzyme activity assay and an enzyme-linked immunosorbant assay (ELISA) for protein quantification. Protein extracts, prepared per manufacturer's protocol in Reporter Lysis Buffer (Promega), were used for the CAT activity assay and the β-galactosidase ELISA assay (Promega). For the radioactive CAT enzyme assay, cell lysates were prepared with chloramphenicol (50 μM final concentration) and ^14^C-butyryl-Coenzyme A (8.3 μCi/mmol final concentration) (Moravek Biochemicals, Brea, CA) and added to 4 ml Econofluor II scintillation fluid (PerkinElmer, Wellesley, MA) (5 ml total volume) as described by Neumann et al [47]. The radioactivity transferred to chloramphenicol by CAT was counted for 0.5 min/tube, and counts were repeated at 45 min intervals during the assay period until counts plateaued. Commercial CAT enzyme (Promega) served as a positive control. CAT activity, as determined by subtracting the activity of mock transfected samples (no DNA added), was normalized to β-galactosidase activity. CAT and β-galactosidase proteins were quantified using ELISA assays (Roche Applied Science, Indianapolis, IN) according to the manufacturer's instructions. CAT protein was normalized to β-galactosidase protein. Both methods for evaluating reporter protein expression were performed in duplicate and repeated a minimum of three times. The Student's T-test was used to analyze CAT activity levels between the pGEM-GGG -44 G construct and the other 5' UTR -44 C containing constructs. The Wilcoxon Rank-Sum test was used to analyze the CAT protein concentrations due to non-normal data distribution.

### RNA extraction and real time quantitative PCR (QPCR)

Total RNA was extracted from HOK cells using the RNeasy Mini Kit (Qiagen, Valencia, CA). Reverse transcription was performed with 500 ng HOK RNA using the RETROscript kit (Ambion, Austin, TX) with oligo(dT) primers according to the manufacturer's protocol. The reaction mix contained 250 nM oligo(dT)primer, 10 mM deoxynucleoside triphosphate mix, 50 U of reverse transcriptase and 13 U of RNase inhibitor, and buffer. QPCR amplification of the resulting cDNA was carried out for hBD1, hBD2, hBD3, beta-actin, and the housekeeping gene ribophosphoprotein, RPO. Table 1 lists primers used. QPCR was performed in a MyiQ iCycler (Bio-Rad, Hercules, CA) using the iQ SYBR^® ^Green Super Mix (Bio-Rad). Melt curve analysis confirmed that the signal was generated by the expected amplification product. The threshold cycles for detection level of hBD1, hBD2, hBD3, and beta-actin were normalized to the housekeeping gene RPO and compared among cell lines with three genotypes of the -44 SNP (CC, CG, and GG). The relative fold change was calculated by the Pfaffl method [48]. Because this method requires a standard for comparison, we used a reference consisting of mRNA pooled from 4 HOK donors randomly selected and combined as a baseline for the entire study. Results are expressed as the relative fold change for each mRNA from individual genotypes compared to this combined RNA reference baseline. Messenger RNA expression data were log-transformed due to non-normal data distribution.

### Antimicrobial assays

Epithelial cell protein extracts for ELISA and microbial assays were prepared from triplicate post-confluent cell cultures grown in 24-well plates. Cultures were washed with PBS and extracted with ice cold extraction buffer (50 mM Tris, pH 7.4, 5 mM EGTA, 150 mM NaCl, 1% Triton X-100 containing protease inhibitors (1 mini-protease inhibitor tablet (Roche Applied Science) per 7 ml buffer) using a total of 100 μl and stored in aliquots. Total HOK protein was measured using the BCA assay (Pierce Biotechnology, Inc. Rockford, IL).

hBD-3 peptide in HOK extracts was assayed by ELISA (PeproTech, Rocky Hill, NJ) using rabbit anti-hBD-3 according to manufacturer's procedure. Protein extracts were diluted 1/20 and standard hBD-3 peptide (PeproTech) was diluted so that the buffer concentration was equivalent in sample and standard peptide dilutions.

Radial diffusion assay for inhibition of bacterial growth was as modified from the procedure described by Lehrer and coworkers [49]. Briefly, Gram positive *Staphylococcus epidermidis *UW3 [50] was grown to mid-log phase and centrifuged, washed, and resuspended in 10 mM phosphate buffer, pH 7.4. Bacteria (4 × 10^6^) were added to 10 ml 1% LMP agarose (Invitrogen) containing 1% Todd Hewitt broth, 10 mM phosphate buffer, pH 7.4 and poured into a square Petri dish. Three mm wells were punched and 4 μl control peptide or HOK protein extract containing 4 μg protein added per well. Plates were incubated for 3 hr at 37°C to allow sample to be absorbed into the agarose, then ten ml nutrient overlay agarose (1% agarose with 200% Todd Hewitt broth) was added and the plates incubated at 37°C overnight. Plates were fixed with 10 ml 5% acetic acid/25% methanol solution, digitized images were obtained using a CCD camera (Alpha Innotech, San Leandro, CA), and the circle of clearing measured using NIH ImageJ 1.38 u software . Measurements were done in a blinded fashion. Triplicate HOK protein extracts were tested in duplicate. Results were analyzed by comparing the area of the zone of clearing minus the area of the well for each sample.

### Statistical analysis

Differences in mRNA expression and antimicrobial activity by radial diffusion assay among the three -44 SNP genotype groups were analyzed one-way analysis of variance (ANOVA). Further testing was performed on individual groups by the Dunnett T3 method [51] which does not assume equal variances. For all comparisons p ≤ 0.05 was considered statistically significant.

## Results

### The -44 SNP affects the predicted structure of the *DEFB1 *5' UTR

**Figure 1:**
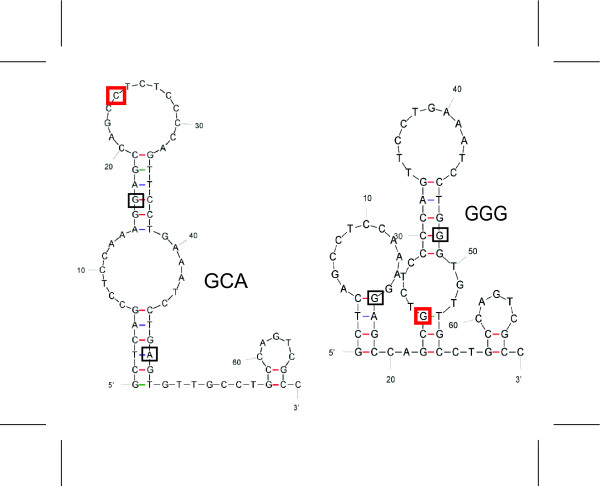
**Putative secondary structure for the 5' UTR of *DEFB1 *mRNA**. Two of the four haplotypes (-52, -44, -20 SNP sites) used in the CAT constructs as calculated and drawn by the MFOLD Quikfold program. The locations of the SNP sites are boxed and the -44 SNP sites are shown in red boxes. Note that the -44 SNP site in the GGG haplotype is in a hybridized stem structure. In contrast, as represented by the GCA structure shown, the -44 SNP site is in a loop or non-hybridized configuration in the other three haplotypes. Similar putative structures were obtained in three separate trials. Secondary structures with the lowest ΔG (G = Gibbs free energy) were evaluated. The ΔG values are: GCA = -13.5, GCG = -12.8, ACG = -10.5, GGG = -13.9.

The *DEFB1 *5' UTR contains the -44 SNP site (rs1800972) as well as two additional SNPs (-20, rs11362 and -52, rs1799946). To see if these SNPs have an effect on the mRNA secondary structure the predicted folding pattern and Gibbs free energy were computed by MFOLD [44] for hBD1 mRNA with common sequence variants or haplotypes (Figure 1). The putative secondary structure for the *DEFB1 *5' UTR containing the two most common haplotypes, GCA (-52,-44,-20)(Figure 1) and ACG (not shown), show the -44 site on exposed loops, but this site is buried in the naturally occurring GGG haplotype which has the -44 G allele (Figure 1). The site is also on an exposed loop in the GCG haplotype (not shown), which was designed as a direct control for the GGG haplotype sequence, but was not observed in the populations analyzed in our laboratory. These putative structural characteristics led us to test the *DEFB1 *5' UTR for effects on reporter protein expression in a model system

### The -44 SNP G allele results in increased reporter protein expression in vitro

**Figure 2:**
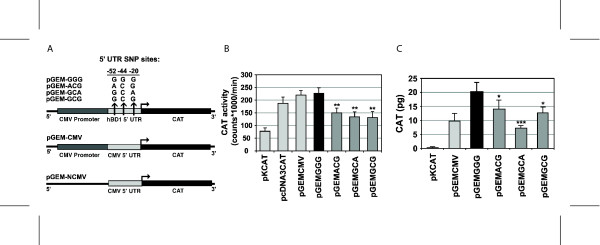
**Effect of the 5' UTR haplotype on reporter protein expression**. A. Schematic representation of reporter gene constructs; details of construction provided in Methods and Additional file 1. Arrows indicate SNP sites in hBD1 5' UTR. Bent arrows indicate direction of translation. Light grey bars represent 5' UTR. Construct names are given at left, the SNP haplotypes comprise the latter portion of the name. B. and C. CAT expression in COS-7 cells transiently transfected with constructs containing hBD1 (or CMV) 5' UTR. CAT expression is normalized to β-galactosidase. (B) CAT activity measured by radioactive enzyme activity assay. Mock transfected (no DNA added) CAT values were subtracted and CAT activity was normalized to β-galactosidase activity obtained by ELISA enzyme activity assay. (C) The quantity of CAT protein was measured by an antibody-based ELISA and normalized to β-galactosidase protein. Black bars in B and C represent constructs containing hBD1 5' UTR with the -44 G allele. Values given are averages and standard errors from 3 or more experiments. Statistically significant differences are indicated with asterisks compared to pGEM-GGG. *, p < 0.05; ** p < 0.01; *** p < 0.001.

The effect of the *DEFB1 *-44 G allele on gene expression was evaluated in COS-7 cells using constructs containing different haplotypes of the *DEFB1 *5' UTR fused to the CAT reporter gene (Figure 2A). Two different assays showed greater CAT expression for the pGEM-GGG construct carrying the -44 G allele. CAT enzyme activity was 1.7 fold (p < 0.01) greater with pGEM-GGG CAT than that observed with pGEM-GCA and pGEM-GCG. It was also greater (1.5 fold, p = 0.01) than the pGEM-ACG construct (Figure 2B). Similarly, CAT protein expression as measured by ELISA showed that pGEM-GGG had the highest expression in COS-7 cells compared to pGEM-GCA (2.8 fold greater, p = 0.001), pGEM-GCG (1.6 fold greater, p = 0.036) and pGEM-ACG (1.5 fold greater, p = 0.047) (Figure 2C). Hence, by both assays, CAT protein expression is significantly increased in cells expressing the -44 G allele in the context of the naturally observed 5' UTR haplotype, GGG, compared to the two most common haplotypes with the -44 C allele, ACG and GCA. Reporter protein expression in cells transfected with the GGG haplotype was also significantly higher than the GCG construct, the direct 5' UTR control, demonstrating that the -44 G allele alone influences reporter gene expression.

### Genotyping of primary human keratinocytes and association of the -44 SNP with hBD1 mRNA expression

**Figure 3:**
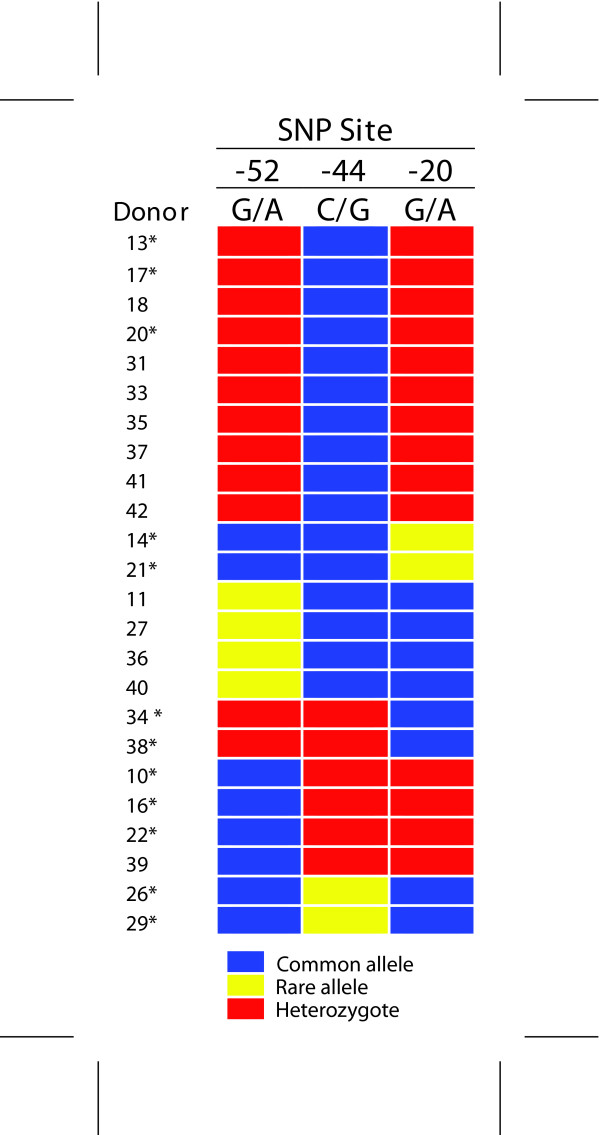
**Genotypes at *DEFB1 *5'UTR SNP sites from 24 keratinocyte donors**. Those samples that were used for further studies are indicated by *.

To examine the effect of the *DEFB1 *-44 SNP in the context of the whole gene, human keratinocytes (HOK) from twenty-four independent donors were evaluated for the *DEFB1 *exon 1 sequence. Sixteen cell lines were homozygous at the -44 site for the common allele, C; six were heterozygous at this site; and two were homozygous for the -44 G allele (Figure 3). The allele frequency observed for the -44 G allele (0.21) is close to that previously reported [30,31]. Frequency of the GG genotype in the population is predicted to be 4% based on Hardy Weinberg equilibrium, thus we were fortunate to identify the GG genotype in two donors. All HOKs with one or more copies with the -44 G allele had a genotype consistent with the GGG haplotype for the -52, -44, -20 SNPs in accordance with our previous report [31].

**Figure 4:**
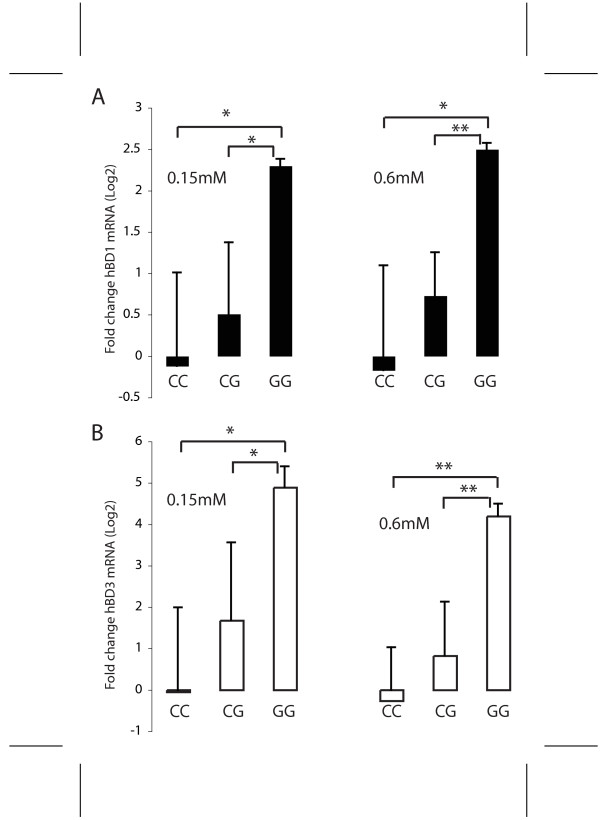
**Association of constitutive antimicrobial gene expression with the *DEFB1 *-44 SNP genotype**. cDNA was made from primary keratinocytes from 12 donors grown in 0.15 mM Ca^2+ ^(left) or 0.6 mM Ca^2+ ^(right). QPCR amplification of cDNA for hBD1(A) and hBD3 (B) and the housekeeping gene RPO was performed. All data were normalized to the housekeeping gene and compared to a separate reference cDNA. The data presented are log2 transformed and represent 3 replicates for each cell line with duplicate QPCR reactions. The mean and std. dev. for each group is shown. *, p < 0.05; ** p < 0.01.

Twelve of the HOK donor lines representing the three -44 genotypes, 5 CC, 5 CG and 2 GG, were further evaluated for mRNA and antimicrobial activity. Given the small sample size available and low power to show differences between groups, the statistical tests were not expected to give definitive results. Nevertheless, because each donor cell line was analyzed in triplicate and some differences were large, we were able to demonstrate statistically significant differences among the groups for several variables. HBD1 mRNA expression was significantly greater in those cells with the GG genotype compared to the homozygous common allele C in HOKs cultured in both 0.15 mM (p = 0.021) and 0.6 mM calcium (p = 0.023) and to the heterozygous CG genotype (p = 0.024; p = 0.003 in 0.15 mM and 0.6 mM calcium) (Figure 4A). Thus, although our sample of the GG genotype is small, cells with this genotype have significantly greater constitutive hBD1 mRNA expression. In contrast, beta-actin mRNA levels were not correlated with genotype (not shown).

### Effects of the -44 SNP on the constitutive level of hBD2 and hBD3

The level of hBD2 and hBD3 mRNAs were determined to identify possible effects of the *DEFB1 *-44 SNP on other β-defensin genes in the chromosome 8p defensin cluster. Because the cultures were not exposed to cytokines or bacterial products, these values represent the constitutive level of the mRNAs. HBD2 mRNA expression was not significantly correlated with the *DEFB1 *-44 SNP in either growth condition (p = 0.570, p = 0.577) (not shown). Surprisingly, we found a significant correlation with the *DEFB1 *-44 SNP and expression of hBD3 mRNA in both growth conditions (Figure 4B). The expression of hBD3 increased with the -44 G allele (CC<CG<GG). HOKs with the GG genotype grown in 0.15 mM calcium had a mean level of hBD3 mRNA 18-fold greater than those with the CC genotype (p = 0.011). In 0.6 mM calcium HOKs with the GG genotype had 16-fold greater relative expression of hBD3 than those with the CC genotype (p = 0.002). In both growth conditions hBD3 mRNA expression in the GG homozygous group was also significantly different from the heterozygous CG genotype group. These results suggest that the -44 G allele in *DEFB1 *is associated with increased hBD3 mRNA expression.

We also evaluated the effect of the *DEFB1 *-44 SNP on hBD3 peptide expression by ELISA (effective reagents were not available for hBD1 and hBD2). HOKs with the GG genotype had a median level of hBD3 peptide two-fold greater than those with the CC genotype (ANOVA p = 0.014), but this was not statistically significant in further analysis accounting for unequal variances between groups due to the small sample size (not shown).

### Antimicrobial activity is increased in HOKs with the -44 G allele

**Figure 5:**
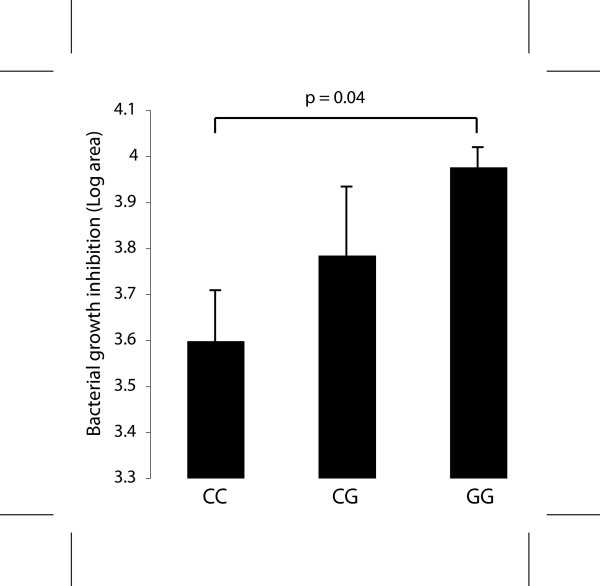
**Association of the *DEFB1 *-44 SNP genotype with increased constitutive antimicrobial activity**. Total cell lysates prepared from cultured keratinocytes (4 μg protein) were added to solidified agarose containing *S. epidermidis*. The area of clearing in square mm. minus the area of the well was calculated and results compared by genotype. The data represent 3 replicates from each cell line and the assay was performed in duplicate. The data are log transformed and the mean and std. dev. are shown.

A radial diffusion assay was used to test for overall antimicrobial activity of HOK extracts using *Staphylococcus epidermidis *UW3, a Gram positive commensal skin organism which has been previously demonstrated to be susceptible to hBD1 (our unpublished findings) as well as hBD3 [52]. The mean area of clearing increased with the -44 G allele (CC<CG<GG) and extracts from cells with the GG genotype showed significantly greater zones of clearing than those with the CC genotype (p = 0.04) (Figure 5).

### Interferon-γ induced levels of hBD1 and hBD3 mRNA are correlated with genotype

**Figure 6:**
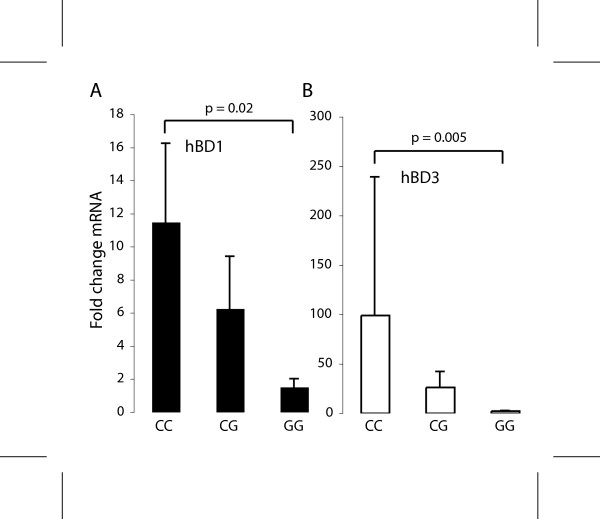
**Association of the *DEFB1 *-44 SNP genotype with hBD1 and hBD3 mRNA expression in HOKs induced with IFNγ**. QPCR was conducted as in Figure 4. Log transformed mean and std. dev. are shown. Note increased hBD1 and hBD3 mRNA in the CC genotype. The data represent 2–3 replicates from each cell line with duplicate PCR assays.

Induced levels of hBD1, hBD2, and hBD3 were evaluated in HOKs from the twelve donors (5 CC, 5 CG, 2 GG) in the presence of LPS and PAM3CSK4, ligands for TLR4 and TLR2, respectively, as well as, IL1β, TNFα, and IFNγ. HBD1, hBD2 and hBD3 mRNA were not upregulated by LPS or PAM3CSK4, as anticipated. TNFα and IL1β induced hBD2 in highly variable amounts in different cell donors, not correlated with genotype, but had little effect of hBD1 and hBD3 mRNA (not shown). In contrast, IFNγ stimulation resulted in significant upregulation of both hBD1 (p = 0.02) and hBD3 mRNA (p = 0.005) that was correlated with genotype with the common -44 CC genotype averaging 12-fold and 100-fold increase over uninduced levels, respectively (Figure 6).

## Discussion

Antimicrobial peptides play an important role as part of mucosal and skin innate immunity. Therefore, genetic variation that results in altered peptide expression may influence susceptibility to infection. Here, we demonstrate that the *DEFB1 *-44 G allele is associated with increased constitutive expression of both hBD1 and hBD3 mRNA, with increased antimicrobial activity in oral keratinocytes, and with increased reporter protein expression in transfected cells, suggesting that the GG genotype results in enhanced transcription of the *DEFB1 *gene or with enhanced post-transcriptional events. We also demonstrate the first evidence of a significant relationship between the -44 SNP in the *DEFB1 *gene with expression of *DEFB103 *gene encoding hBD3 in the nearby gene cluster. The antimicrobial functional assay also supports greater peptide expression in keratinocyte extracts. This assay is not specific for hBD1 but could be the result of increased expression of other defensins including an increased level of hBD3 peptide. Results from our model system as well as the keratinocyte expression data demonstrate that the *DEFB1 *SNP -44 G allele is associated with increased constitutive antimicrobial peptide expression and function. Thus, within the context of the β-defensin genes, the -44 SNP is associated with a direct *cis *effect on the expression of hBD1 and an indirect effect on hBD3 expression. Together, these findings suggest a mechanism which supports genetic studies that have demonstrated a correlation between the *DEFB1 *5' UTR -44 SNP G allele and protection from various types of infection [34-36,39].

The approach of examining transcript and reporter protein expression in a model system is designed to investigate direct *cis *effects of the *DEFB1 *UTR. Using an in vitro system in which we tested the expression of different *DEFB1 *5' UTRs fused to the CAT reporter gene, we have shown that the -44 G allele is specifically associated with increased protein expression. While our studies agree with previous findings of a 2.3 fold increase of luciferase expression with the -44 G allele compared to the more common C allele in a short promoter construct [33], Milanese and coworkers [53] reported a 53% decrease in transcription with the -44 G construct. These differences may be related to a possible role of the SNP in post-transcriptional regulation or mRNA stability that varies with cell type. The difference in predicted mRNA structure for the *DEFB1 *5' UTR between the -44 genotypes, the highly conserved nature of the *DEFB1 *5' UTR in primates [54] and the length of this region (71 nucleotides), considerably longer than that of *DEFB4 *(36 nucleotides) and *DEFB103 *(30 nucleotides), are consistent with a possible role for the 5' UTR in post-transcriptional control via RNA-binding domains [55].

In physiological conditions, the level of β-defensin expression in many tissues varies with exposure to bacteria and proinflammatory cytokines. Thus it was of interest to examine induced levels of these antimicrobial peptides. A significant correlation of hBD1 and hBD3 mRNA with genotype was not seen for proinflammatory cytokines IL1β and TNFα, and although hBD2 was highly upregulated by these stimulants, expression was not associated with the -44 *DEFB1 *SNP genotype. HOK expression of these three β-defensins was not induced by TLR2 and TLR4 ligands, in agreement with previous results for this cell type which is hyporesponsive to purified ligands, but responds to more complex bacterial signals [11,12,56]. In contrast to constitutive levels, HOKs with the more common CC genotype had increased mRNA levels for hBD1 and hBD3 in the presence of IFNγ. Induction of hBD1 and hBD3 by IFNγ was anticipated [57,58], but the association of hBD3 upregulation with *DEFB1 *SNP genotype was quite unexpected. This result suggests the possibility of linkage disequilibrium of the *DEFB1 *-44 SNP and a *DEFB103 *promoter site.

Genetic variation that affects level of expression is generally associated with transcription factor binding sites in the promoter region of genes, however, the *DEFB4 *and *DEFB103 *genes, encoding hBD2 and hBD3, respectively, also exhibit copy number polymorphism [26,59] which affects expression levels. Copy number polymorphisms are frequently in linkage disequilibrium with SNPs in nearby genes [60]. Linkage disequilibrium of the -44 SNP G allele of *DEFB1 *with a high copy number of the *DEFB103 *gene may be the explanation for the increased constitutive expression hBD3, while the *DEFB1 *-44 C allele may be associated with a *DEFB103 *promoter region alteration that affects IFNγ inducibility. Investigation of these possibilities is beyond the scope of the present work, nevertheless, it seems clear that a high constitutive level of hBD1 and hBD3 expression may be beneficial in some situations while the greater IFNγ inducibility may be beneficial in others.

Our findings have biological relevance in understanding mechanisms that may be associated with a disease susceptibility risk that correlates with the -44 SNP G allele. Several factors contribute to a protective function including direct effects resulting in increased constitutive hBD1 and hBD3 antimicrobial activity and increased immunomodulatory effects of these defensins. Higher constitutive level of hBD1 and hBD3 in individuals with the -44 G allele is consistent with the protection from oral *Candida *carriage [34] and may be biologically important to enhance resistance of the epithelium to *Candida *colonization because hBD3 has strong anti-candidal activity [57,61,62]. Similarly, the protection from chronic obstructive pulmonary disease [35] and lower risk of HIV-1 infection in children born to HIV-1 positive mothers (Braida, 2004) associated with the -44 G allele may be related to increased expression of hBD3 which has more potent direct antimicrobial activity than hBD1 with has direct anti-viral properties by blocking viral replication [63] and acting as an antagonist of the HIV-1 co-receptor, CXCR4 [64].

On the other hand, the more common C allele of the *DEFB1 *-44 SNP results in greater levels of hBD1 and hBD3 in the presence of IFNγ. Therefore, in pathological conditions in which T helper cell type 1 immune response is elicited, the CC genotype may be protective. This includes periodontitis which has been considered a Th1 type condition [65-67] with elevated levels of IFNγ in gingival fluid of active periodontal lesions [68,69]. Periodontal pathogens like *P. gingivalis *and *Aggregatibacter actinomycetemcomitans *induce the secretion of IFNγ from leukocytes and T-cells, respectively [70,71]. Thus, IFNγ not only exhibits pro-inflammatory characteristics during progression of active periodontal lesions, but may also promote protective capabilities in the early stage of infection by increasing the gene expression of hBD1 and hBD3 in HOKs carrying the common -44 CC genotype. Because hBD1 and hBD3 are highly expressed in the -44 GG genotype, it may be that induction by IFNγ remains unapparent for this genotype, whereas the common -44 CC genotype with low baseline expression of hBD1 and hBD3 could allow at least a short term protective effect by IFNγ. Therefore, perhaps it is not surprising that the -44 SNP has not been associated with either early onset [72] or with severe chronic periodontal disease [73].

## Conclusion

The G allele of the -44 *DEFB1 *SNP is associated with higher constitutive levels of both hBD1 and hBD3 in keratinocytes in a manner that is consistent with protection from oral candidiasis. In contrast, the more common C allele is associated with greater hBD1 and hBD3 expression in IFNγ-stimulated keratinocytes and is likely to be protective in conditions with elevated IFNγ such as chronic periodontitis. The contrast of constitutive vs. induced levels of hBD1 and hBD3 will make interpretation of genetic studies for the *DEFB1 *-44 SNP locus extremely complex.

## Abbreviations

hBD: human beta-defensin; SNP: single nucleotide polymorphism; UTR: untranslated region; HOK: human oral keratinocytes; KBM: keratinocyte basal medium; CAT: chloramphenicol acetyltransferase; QPCR: quantitative real time PCR.

## Competing interests

The authors declare that they have no competing interests.

## Authors' contributions

AK carried out the mRNA extraction and cDNA preparation, ELISA analyses, helped design and interpret the study, and drafted the manuscript; LPF conducted the plasmid preparation and reporter construct assays; BH isolated and cultured HOKs from various donors and conducted and analyzed the quantitative PCR for the induced cultures; HD carried out the quantitative PCR and performed initial statistical analysis; RP designed the plasmid construction and approach; JK conducted the antimicrobial assays and DNA preparation for genotyping; BD was responsible for the overall experimental design and coordination and helped to draft the manuscript. All authors read and approved the final manuscript.

## Pre-publication history

The pre-publication history for this paper can be accessed here:



## Supplementary Material

Additional file 1 **Preparation of plasmids**. Detailed methods for preparation of the 5' UTR CAT constructs. Click here for file
