# Supplementary material for: Association of a genetic polymorphism (-44 C/G SNP) in the human DEFB1 gene with expression and inducibility of multiple β-defensins in gingival keratinocytes
Source: BMC Oral Health. 2009 Aug 27;9:21. doi: 10.1186/1472-6831-9-21 (PMC2739845; doi:10.1186/1472-6831-9-21)
Supplement: Additional file 1 — Preparation of plasmids. Detailed methods for preparation of the 5' UTR CAT constructs. [file 1472-6831-9-21-S1.doc]

Association of a genetic polymorphism (-44 C/G SNP) in the human *DEFB1* gene with expression and inducibility of multiple β-defensins in gingival keratinocytes

**Additional file 1 – Preparation of plasmids**

*Plasmid constructs*

We prepared four constructs containing the most common haplotypes of the three known hBD-1 5’ UTR SNPs (-20, -44, -52) (Jurevic, Chrisman et al. 2002) (GenBank accession no. U50930; rs 11362, 1800972, 1799946) upstream of the chloramphenicol acetyltransferase (CAT) reporter gene using the pc DNA3CAT plasmid (Invitrogen, Carlesbad, CA). Details of plasmid construction are included in the Supplementary file. A construct with the CMV 5’ UTR and a promoterless pGEM-NCMV were created and served as controls. Table 1 lists all primers used. In brief, after removal of the Kpn I to Apa I region from the pcDNA3CAT plasmid (Invitrogen, Carlesbad, CA), the CAT-poly A region was amplified with the common downstream primer BamCATbGH and either the ClaCAThBD-1 or ClaCATCMV upstream primers, for the hBD-1 or CMV 5’UTR-containing constructs, respectively. These products were placed into pGEM7Zf+ plasmids (Promega, Madison, WI). The 5’ regions containing the basal promoter (TATA box) and 5’ UTR were amplified from pcDNA3CAT using the KpnCMV and XcmICMV primers or from human genomic DNA using the KpnhBD-1 and XcmIhBD-1 primers, and added upstream to the CAT gene in the pGEM7Zf+ construct. The CMV promoter was amplified from pcDNA3CAT using the KpnCMV2 and XhoCMV primers and added upstream of the 5’ UTR in the pGEM7Zf+ constructs. Finally site-directed mutagenesis (Stratagene (La Jolla, CA) QuickChange Site-Directed Mutagenesis kit) was employed to correct three bases in the hBD-1 5’ UTR (that formed part of a restriction site used for cloning) to the published wild type sequence. The same mutagenesis approach was used to change the wild type bases at the three 5’ UTR SNP sites to match the desired haplotypes. The constructs with the common haplotypes were: pGEM-ACG (‑20=A, -44=C, -52=G), pGEM-GCA and pGEM-GGG; an additional construct, pGEM-GCG, was also prepared as a direct control for pGEM-GGG, even though this haplotype has not been observed in the general population{Jurevic, 2002 #2}.

**Table 1S.** Oligonucleotide PCR primers used for plasmid construction. All sequences are shown 5’ to 3’.

| **Name** | **Sequence** | **Anneal. Temp.** |
| --- | --- | --- |
| **Cloning** |  |  |
| BAM-CATBGH | CCCGGATCCCCAGCATGCCTGCTATTGTC |  |
| CLA-CAT-CMV | CCCATCGATCCAAAGCTAAAATGGAGAAAAAAATCACTGGATATACC |  |
| CLA-CAT-HBD1 | CCCATCGATCCACAGTCGCCATGGAGAAAAAAATCACTGGATATACC |  |
| CMVPromF | GTGCTGCAAGGCGATTAAGT |  |
| HBD1 XCM UTR | CAAAATGTTCTTTACGATGC |  |
| HTATARev | CCGGATGAGCATTCATCAGGCGGGCAAG |  |
| KPN-CMV | CCCGGTACCTCTATATAAGCAGAGCTCTCTG |  |
| KPN-CMV2 | CCCGGTACCACCGTACACGCCTACCGCC |  |
| KPN-HBD1 | CCCGGTACCAGTCTTATAAATACAGTGACGC |  |
| XCM1-HBD1 | CCCCCATGGCGACTGTGGGGCAACAC |  |
| XHO-CMV | CCCCTCGAGCGATGTACGGGCCAGATATACG |  |
|  |  |  |
| **Mutagenesis** |  |  |
| hBD1 Mut SeqF | GGTAGGCGTGTACGGTGGTA | 55 |
| HBD6608ACF | GCTCAGCCTCCAAAGAAGCCAGCCTCTCCCCAGTTCC | 55 |
| HBD6608GCF | GCTCAGCCTCCAAAGGAGCCAGCCTCTCCCCAGTTCC | 55 |
| HBD6608GG2 | CAGGAACTGGGGAGACGCTGGCTCCTTTGGAGGC | 55 |
| HBD6608GGF2 | GCCTCCAAAGGAGCCAGCGTCTCCCCAGTTCCTG | 55 |
| HBD660A8C | GGAACTGGGGAGAGGCTGGCTTCTTTGGAGGCTGAGC | 55 |
| HBD660G8C | GGAACTGGGGAGAGGCTGGCTCCTTTGGAGGCTGAGC | 55 |
| HBD692A | CCATGGCGACTGGCAGGCAACACTCAGGATTTCAGG | 55 |
| HBD692AF | CCTGAAATCCTGAGTGTTGCCTGCCAGTCGCCATGG | 55 |
| HBD692G | CCATGGCGACTGGCAGGCAACACCCAGGATTTCAGG | 55 |
| HBD692GF | CCTGAAATCCTGGGTGTTGCCTGCCAGTCGCCATGG | 55 |
|  |  |  |
